# Supplementary material for: Global deletion of Malat1 alters alcohol consumption in a sex-specific manner
Source: bioRxiv. 2026 Jun 24:2026.06.19.733448. Preprint. [Version 1] doi: 10.64898/2026.06.19.733448 (PMC13320743; doi:10.64898/2026.06.19.733448)
Supplement: Supplement 1 [file media-1.pdf]

Supplementary Table 1. gRNA target Sequences

Name	Sequence
Malat1 TAKO gRNA1	TCCTCCCAGGAAAACGCAAA AGG
Malat1 TAKO gRNA2	AAAGGGACACGTCACTCCAC CGG
Malat1 TAKO gRNA3	AACTTATCTGCGATTTCCTC GGG
Malat1 TAKO gRNA4	GTTTAGGAGATTGTAAAGGG AGG
Malat1 5' gRNA	GTTCTCTAGAAATATTCCCG TGG
Malat1 3' gRNA	GGGTGTAAGGCTTGATTGAG TGG

Underlined sequence indicates the protospacer adjacent motif

Supplementary Table 2. PCR Primer Sequences

Name	Sequence	Amplicon Size
Malat1 Promoter F	CTCCATCTTGTTTCGCA	WT- 933bp
Malat1 Promoter R	AAGTAGGTTAAGTTGACGGCC	
Malat1 qPCR F	GGCGGAATTGCTGGTAGTTT	197bp
Malat1 qPCR R	AAGGCGTGTACTGCTATGCT	
Malat1 5' loxP F	TTGGAAAAGACCCACGAAACAA	WT- 375bp<br>loxP 413bp
Malat1 5' loxP R	ACTTTAGGGGGCGAGGGAAG	
Malat1 3' loxP F	TGCAATACTGTGTGTAAGTGTGC	WT- 415bp<br>loxP 453
Malat1 3' loxP R	CAGGTGAGCAAAATGGTCTCC	
Malat1 ReA F	GGGGAAGACAGTGGGCATTT	Unrecombined: 10.5KB<br>Recombined: 150bp
Malat1 ReA R	TACACCCTGGGCAAAAACATC	
β-Actin F	GACCTCTATGCCAACACAGT	150bp
β-Actin R	AGTACTTGCGCTCAGGAGGA	
